# Improving the Stability of Astaxanthin by Microencapsulation in Calcium Alginate Beads

**DOI:** 10.1371/journal.pone.0153685

**Published:** 2016-04-19

**Authors:** Shen-Fu Lin, Ying-Chen Chen, Ray-Neng Chen, Ling-Chun Chen, Hsiu-O Ho, Yu-Han Tsung, Ming-Thau Sheu, Der-Zen Liu

**Affiliations:** 1 Graduate Institute of Biomedical Materials and Tissue Engineering, College of Biomedical Engineering Taipei Medical University, Taipei, Taiwan (ROC); 2 School of Pharmacy, College of Pharmacy, Taipei Medical University, Taipei, Taiwan (ROC); 3 Department of Cosmetic Science and Management, Mackay Medicine, Nursing and Management College, Taipei, Taiwan (ROC); 4 Center for General Education, Hsuan Chuang University, Hsinchu, Taiwan (ROC); National Central University, TAIWAN

## Abstract

There has been considerable interest in the biological functions of astaxanthin and its potential applications in the nutraceutical, cosmetics, food, and feed industries in recent years. However, the unstable structure of astaxanthin considerably limits its application. Therefore, this study reports the encapsulation of astaxanthin in calcium alginate beads using the extrusion method to improve its stability. This study also evaluates the stability of the encapsulated astaxanthin under different storage conditions. The evaluation of astaxanthin stability under various environmental factors reveals that temperature is the most influential environmental factor in astaxanthin degradation. Stability analysis shows that, regardless of the formulation used, the content of astaxanthin encapsulated in alginate beads remains above 90% of the original amount after 21 days of storage at 25°C. These results suggest that the proposed technique is a promising way to enhance the stability of other sensitive compounds.

## Introduction

There has been considerable interest in the biological functions of carotenoids in preventing degenerative diseases such as atherosclerosis, cancer, and aging. Carotenoids are the most widely distributed class of pigments in nature. Astaxanthin (3,3'-dihydroxy-β,β'-carotene-4,4'-dione) is the main keto-carotenoid found in many marine organisms and birds [1–3]. Because of the structural similarity between carotenoids such as β-carotene, zeaxanthin, and lutein, they share many of the same metabolic and physiological functions [4,5]. However, the presence of the hydroxyl and keto groups on each ionone ring provides astaxanthin with some unique features, including a higher antioxidant activity, a polar configuration, and the ability to be esterified. A number of studies have demonstrated that astaxanthin has much stronger antioxidant activity than vitamin E and β-carotene, and is significantly more effective than β-carotene and lutein at preventing the UV light photooxidation of lipids. In addition, the anti-cancer activity of astaxanthin in mammals has been evaluated. Compared to rats fed with carcinogens only, rats fed with carcinogen but supplemented with astaxanthin revealed a significant decrease in the incidence of induced colon cancer. The protective effect of astaxanthin was even more pronounced than that of β-carotene. The growth-inhibitory effects of the astaxanthin-rich *Haematococcus pluvialis* extracts in HCT-116 colon cancer cells were reported. Astaxanthin inhibits cell growth in a dose- and time-dependent manner by arresting cell cycle progression and promoting apoptosis. The studies above suggest the effects and potential applications of astaxanthin in nutraceutical, cosmetics, food, and feed industries [6–8].

Because of its highly conjugated, double bond structure, astaxanthin is unstable during production and storage. This causes a loss of its biological activity, and considerably limits its use in nutraceutical and cosmetic applications. A number of studies have been conducted in an attempt to improve the stability and solubility of astaxanthin. Apart from synthesis of astaxanthin derivatives with improved solubility or stability, many techniques, such as microencapsulation have been investigated. For example, several studies have used cyclodextrin (CD) on the inclusion of carotenoids. The formation of a β-CD-mediated inclusion complex can improve the physical and chemical properties of carotenoids. Kim et al. prepared water soluble and stable Inclusion complex (IC) of astaxanthin using β-CD [9, 10].

Microencapsulation with polymer matrices has received increasing attention in the last decade, resulting in a great number of applications in industry, agriculture, medicine, pharmacy, and biotechnology. Microencapsulation is a technique in which tiny particles are coated or embedded in a homogeneous or heterogeneous matrix to provide a physical barrier between the core material and environmental conditions. The advantages of applying microencapsulation include reducing the core reactivity with environmental factors, decreasing the transfer rate of the core material to the outside environment, promoting easier handling, controlling the release of the core material, masking the core taste, and diluting the core material when it should be used in only very small amounts [11–13]. Because of the biocompatibility and biodegradability of natural polymers, there are many reports and patents on the use of chitosan in preparing microspheres and microcapsules. Higuera-Ciapara et al. microencapsulated astaxanthin in a chitosan matrix using the multiple emulsion/solvent evaporation method [14]. Kittikaiwan et al. repeatedly immersed *H*. *pluvialis* beads in a chitosan solution to form chitosan-algae microcapsules [15]. Alginate is a linear biopolymer consisting of β-1,4 linked D-mannuronic acid (M block) and L-guluronic acid (G block). The cooperative binding of divalent cations localized between homopolymeric blocks of guluronate residues resulted in the gelling properties of alginate. Because of its physicochemical properties, alginate has been used as a thickening, gel-forming, and encapsulating agent in the food and drug industry [16–18]. The emulsification method is the most widely and typically used method in microencapsulation technique. Other methods are based on emulsion preparation between the drug and the encapsulating matrix, followed by spray drying of the emulsion. The main disadvantage of these methods is that drug losses occur during the process and the resulting product includes organic solvent [19–21]. Compared with the emulsification method, the extrusion method is simple and mild. Therefore, the aim of the current work is to encapsulate astaxanthin in sodium alginate beads using the extrusion method to further increase its stability. This study also evaluates the stability of the encapsulated astaxanthin under different storage conditions in terms of astaxanthin content.

## Materials and Methods

### Materials

Astaxanthin was obtained from Orgchem Technologies (Hsinchu, Taiwan). Sodium alginate was purchased from Sigma-Aldrich (Dorset, UK). Carophyll^®^ Pink 10% CWS was obtained from DSM Nutritional Products Ltd. (Kaiseraugst, Switzerland). Calcium chloride and Tween 20 were purchased from Riedel-de Haën (Seelze, Germany). The HPLC grade analytical reagents used included acetonitrile, dichloromethane (Merck, Germany), methanol (Mallinckrodt, USA) and water.

### Analytical determination of astaxanthin content

The astaxanthin level was determined with a high performance liquid chromatography (HPLC) system with a Gemini C18 110A (5 μm, 150 x 4.60 mm) Waters HPLC column and a mobile phase consisting of 83% methanol, 7% water, 5% acetonitrile, and 5% dichloromethane. Subsequently, 50 μL of sample was injected to HPLC with the temperature kept at 25°C. The detection and quantitative measurement of astaxanthin was performed at 480 nm. The calibration of the peak area versus astaxanthin concentration was linear in the concentration range of 50–10 000 nM (R^2^ = 0.9999, n = 6). Injections were performed in duplicate for each sample and standard. To determine the astaxanthin content in the beads, samples were crushed and the astaxanthin was extracted by dichloromethane and pH 6.8 phosphate buffer repeatedly. The obtained solution was then passed through a 0.22 mm filter before further HPLC analysis.

### Evaluation of astanxanthin stability under various environmental factors

The stability of the astaxanthin was examined under various environmental factors, including light, temperature, and nitrogen gas. Astaxanthin was contained in small glass bottles placed under different environmental conditions. For the samples tested for temperature, bottles were stored at -30°C, 4°C, and 25°C. For the samples tested for exposure to light, control samples were covered with foil paper. For samples subjected to nitrogen exposure, nitrogen was purged into the bottles then secured tightly. Samples were taken at scheduled times (i.e., the 1st, 4th, 7th, 11th and 16th day) to analyze the astaxanthin content.

### Preparation of astaxanthin encapsulated sodium alginate beads

The extrusion method was employed to encapsulate astaxanthin in sodium alginate beads. One percent of astaxanthin was dispersed in various concentrations of sodium alginate solution containing various concentrations of Tween 20. The polymer solution was then added dropwise into 10 mL of calcium chloride solution using a hypodermic syringe through a #27G needle under constant stirring at 150 rpm for 15 min at room temperature. The resulting beads were separated from the solution by centrifugation and then allowed to dry at room temperature in a dust-free chamber. Table 1 lists the various experimental conditions, such as concentration of calcium chloride solution (2% and 10%), sodium alginate solution (1%, 2%, and 3%) and the surfactant Tween 20 (0.5% and, 2%). The particle size and morphology of beads were measured using the built-in function of Colony Count in UVP BioSpectrum AC Imaging System (UVP, Upland, CA, USA). The microencapsulation yield (MY) in the microencapsulation process was calculated as the dry weight gain of bead samples divided by the total weight of sodium alginate, calcium chloride, and astaxanthin used in the process. The loading efficiencies were determined as the ratio of the calculated weight of astaxanthin in the bead samples to the actual weight of astaxanthin used in the process. Experiments were performed in triplicate.

**Table 1:** Preliminary experimental conditions evaluated for the preparation of calcium alginate beads without astaxanthin and results of physical characteristics. The word “ALG” denotes alginate.

**Code name**	CaCl_2_ concn.(%)	Tween 20 in ALG (%)	ALG concn.(%)	Glycerol/H_2_O	Needle (G)	Stirring rate(rpm)	Stirring time(min)	Weight (g)	Weight (%)	Diameter (μm)
**A**	20	0.5	3	50/50	27	150	15	-	-	-
**B**	20	0.5	1	0/100	27	150	15	0.21±0.02	9.1±0.8	728.7±194.3
**C**	10	0.5	1	0/100	27	150	15	0.18±0.02	15.4±1.6	620.3±92.0
**D**	2	0.5	1	0/100	27	150	15	0.15±0.03	45.8±10.3	566.0±92.5
**E**	5	0.5	1	0/100	27	150	15	0.15±0.02	23.7±3.1	601.9±98.3
**F**	2	0.5	2	0/100	27	150	15	0.27±0.02	57.9±4.2	726.8±78.6
**G**	10	0.5	2	0/100	27	150	15	0.41±0.07	31.0±5.8	822.0±100.9
**H**	5	0.5	2	0/100	27	150	15	0.31±0.03	40.6±5.0	749.0±61.1
**I**	10	0.5	1	0/100	24	150	15	0.18±0.01	15.4±0.9	712.8±89.9
**J**	2	0.5	2	0/100	24	150	15	0.27±0.01	59.4±2.0	838.7±66.9
**K**	20	0.5	2	0/100	27	150	15	0.53±0.11	21.6±.3	885.6±112.6
**L**	5	0.5	3	0/100	27	150	15	0.50±0.01	56.0±0.8	903.2±59.0
**M**	2	0.5	3	0/100	24	150	15	0.45±0.02	77.6±2.7	994.5±100.0
**N**	10	0.5	3	0/100	24	150	15	0.63±0.02	43.9±2.3	1029.2±99.7
**O**	10	0.5	3	0/100	24	150	30	0.57±0.04	39.5±2.8	1047.7±121.4
**P**	10	0.5	3	0/100	24	150	60	0.61±0.02	42.4±0.6	1112.9±71.1
**Q**	20	0.5	3	0/100	24	150	15	0.78±0.10	30.2±3.8	1136.9±90.5
**R**	20	0.5	3	0/100	24	150	30	0.77±0.03	29.6±1.0	1171.7±124.0
**S**	20	0.5	3	0/100	27	150	15	0.77±0.07	29.7±2.6	1053.4±99.7
**T**	5	0.5	3	0/100	24	150	15	0.51±0.01	56.9±0.9	1041.3±86.6
**U**	20	0.5	3	0/100	24	150	60	0.84±0.05	32.6±1.9	1156.4±88.9
**V**	5	0	3	0/100	24	150	15	0.50±0.04	55.8±3.8	1199.4±94.0
**W**	20	0	3	0/100	24	150	15	0.75±0.04	29.1±1.5	1326.4±130.8
**X**	5	2	3	0/100	24	150	15	0.55±0.01	62.1±1.2	1026.2±46.2
**Y**	20	2	3	0/100	24	150	15	0.94±0.03	36.2±1.2	1192.5±64.3
**Z**	20	0.5	3	10/90	27	150	15	1.24±0.08	48.2±1.5	1460.2±214.5
**AA**	20	0.5	3	20/80	27	150	15	1.23±0.10	47.6±3.2	1356.3±77.5
**AB**	20	0.5	3	0/100	24	300	30	0.74±0.11	28.7±0.9	1332.2±513.2
**AC**	20	0.5	3	0/100	24	500	30	0.74±0.09	28.7±5.7	1162.4±176.8

### Storage stability

The stability of the astaxanthin in the sodium alginate beads with various formulations was monitored under different storage conditions (25°C, RH 60%, and 40°C, and RH 75%). At specified times (i.e., the 1st, 7th, 14th, and 21st days), 50 mg samples were taken for assay of astaxanthin content. The stability measurement continued for 21 d.

### Statistical analysis

All results were expressed as the mean ± standard deviation of the mean. This study compares the data between treatment groups using one-way analysis of variance (ANOVA). Differences were considered significant at *p* < 0.05.

## Results and Discussion

**Fig 1:**
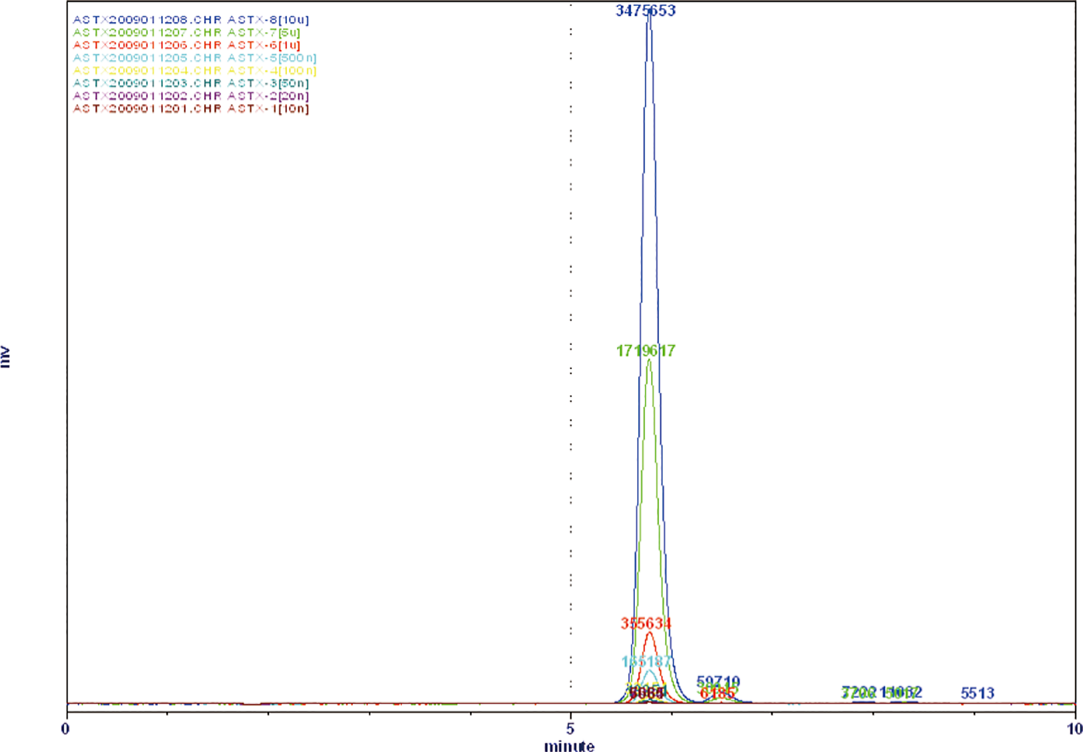
A typical chromatogram of astaxanthin.

**Fig 2:**
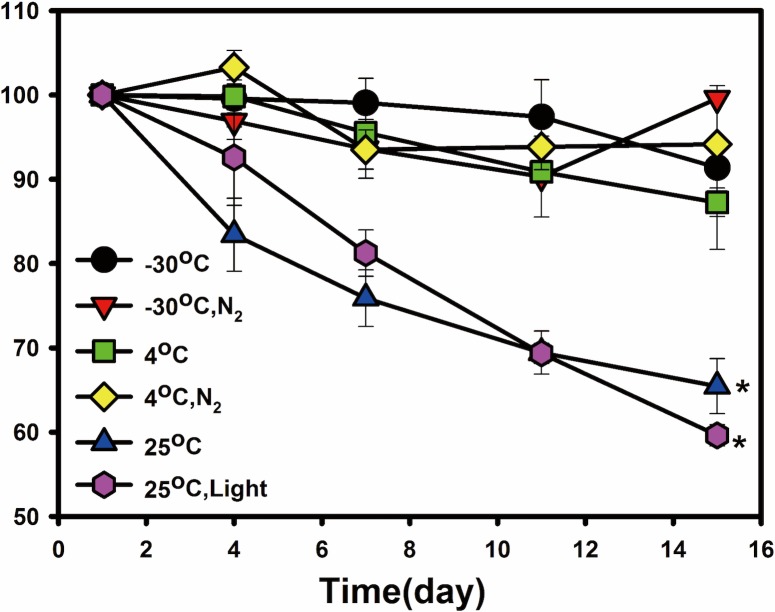
Comparison of the stability of astaxanthin under various environmental factors. The asterisk indicates statistically significant difference (*p* < 0.05).

Fig 1 shows a typical chromatogram for the astaxanthin in this study. The peak in this figure is characterized by a retention time of 5.6 min. The stability of astaxanthin under various environmental conditions was evaluated to determine the effects of the light, temperature, and nitrogen gas. Results show that the minimum loss of astaxanthin, 0.53%, was obtained under a nitrogen atmosphere at -30°C after 16 d. Fig 2 shows that the most influential environmental factor was temperature, as the lowest remaining content of astaxanthin (62.3%) appeared at a storage condition of 25°C after 16 d.

**Fig 3:**
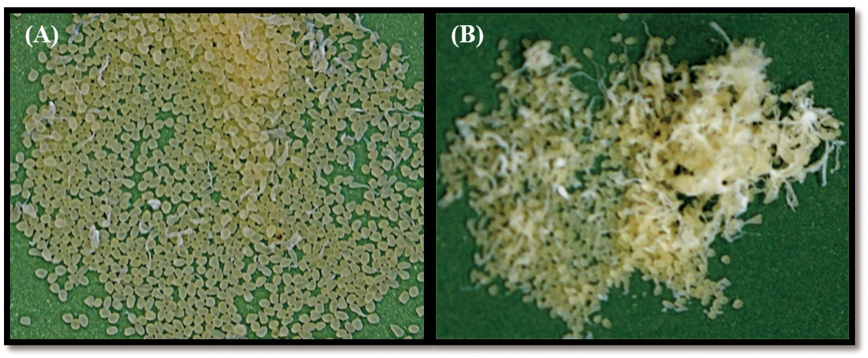
Photo images of alginate beads formed at different stirring rates. The stirring rate set at 150 rpm (A) and 500 rpm (B).

**Fig 4:**
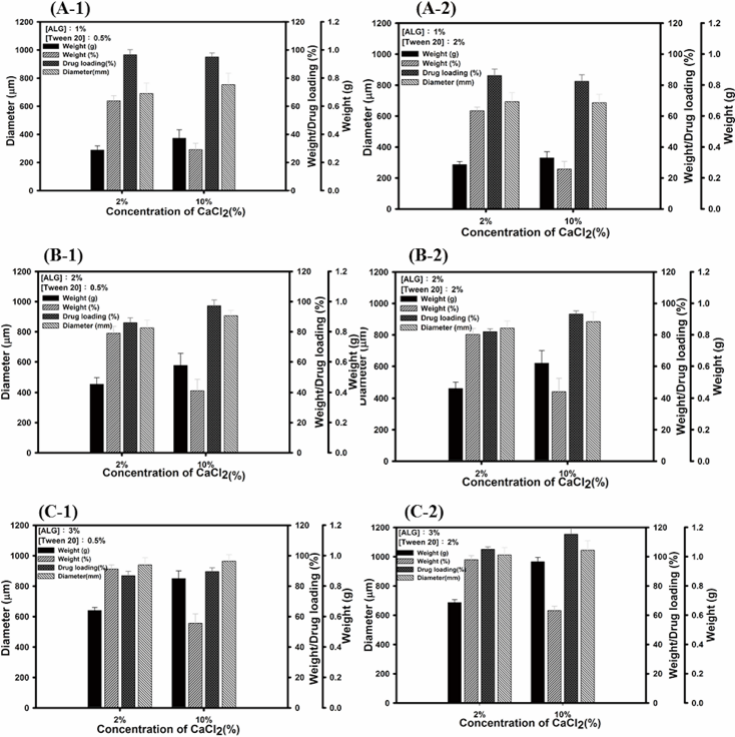
Comparison of astaxanthin encapsulated alginate beads with various formulations with different calcium chloride concentrations (2% and 10%). The word “ALG” denotes alginate. The asterisk indicates statistically significant difference (**✽:** *p* < 0.05, compared to 2%).

The microencapsulation of astaxanthin was carried out by the extrusion method. In this method, spherical beads were obtained by dripping the polymer solution (alginate solution) into the calcium chloride solution. This instantly formed small beads that entrapped drugs within a tridimensional lattice. Preliminary tests summarized in Table 1 revealed the appropriate experimental parameters for preparing alginate beads. A comparison of stirring times while the alginate droplets were dripped in calcium chloride solution revealed no significant difference among treatments (15 min, 30 min, and 60 min) in terms of average size, microencapsulation yield, and loading efficiency. Hence, the stirring time was set at 15 min in this study. The optimum stirring rate was 150 rpm (Fig 3A) because a faster stirring rate resulted in deformed or irregularly-shaped beads (Fig 3B). This study further investigates the ideal experimental conditions for preparing astaxanthin encapsulated sodium alginate beads, including the concentration of calcium chloride solution, sodium alginate solution, and the surfactant Tween 20 (Table 2). Table 3 lists the average yield weight, microencapsulation yield, average size, and loading efficiency of astaxanthin encapsulated beads with various formulations. This table represents each formulation using a code name. For example, the code name “1A0.5T2C” denotes a formulation using 1% of alginate solution, 0.5% of Tween 20, and 2% of calcium chloride solution in the microencapsulation process. The microencapsulation yield and loading efficiency are both key aspects that must be considered in microencapsulation. The microencapsulation yield ranged from 97.94–25.83%, and the loading efficiency ranged from 100% to 82%. Considering the effect of experimental factors in this study, a higher concentration of sodium alginate led to greater average weight and size of alginate beads. This is because the drop size of alginate solution was expected to increase with increasing the viscosity of alginate solution resulting from higher alginate concentration. However, a higher concentration of calcium chloride led to lower microencapsulation yield no matter which alginate concentrations used (Fig 4). This is because calcium ions are capable of binding to the limited carboxylic groups of alginate, leading to the formation of a strong thermostable gel. Once the binding sites were fully occupied, the excess calcium ions were unable to incorporate into alginate beads. Hence, the more calcium chloride not entrapped the less microencapsulation yield would result. On the other hand, the addition of surfactant Tween 20 has no effect on the microencapsulation yield and loading efficiency.

**Table 2:** Optimization of Experimental conditions^*a*^ evaluated for the preparation of calcium alginate beads containing astaxanthin.

Surfactant concentration in ALG solution	ALG* concentration	CaCl_2_ concentration
0.5%	1%	2%
2.0%	2%	10%
	3%	

^***a***^All formulations were injected with a 27 G needle into aqueous solution stirring at 150 rpm for 15 min.

*The word “ALG” denotes alginate.

**Table 3:** Results of physical characteristics including average yield weight, microencapsulation yield, average size, and loading efficiency for astaxanthin encapsulated beads prepared with various formulations.

Code name	Average Yield Weight (g)	Microencapsulation Yield (%)	Average Size (μm)	Loading Efficiency (%)
**1A0.5T2C**	0.29±0.03	63.8±3.6	690.5±74.5	96.6±3.6
**1A0.5T10C**	0.37±0.06	29.2±4.5	754.2±80.7	95.0±2.9
**1A2T2C**	0.29±0.02	63.4±2.4	692.7±58.8	86.2±4.2
**1A2T10C**	0.33±0.04	25.8±4.9	685.9±55.1	82.5±4.2
**2A0.5T2C**	0.46±0.04	79.0±4.6	825.4±52.5	86.0±3.2
**2A0.5T10C**	0.58±0.08	41.1±7.3	906.4±35.6	97.3±3.8
**2A2T2C**	0.46±0.04	80.3±3.8	842.7±45.0	82.0±1.8
**2A2T10C**	0.62±0.08	44.2±8.2	883.6±62.2	93.3±2.0
**3A0.5T2C**	0.64±0.02	91.3±2.6	939.4±47.9	86.8±2.9
**3A0.5T10C**	0.85±0.05	55.7±6.1	964.0±43.0	89.6±2.5
**3A2T2C**	0.69±0.02	97.9±2.8	1011.8±50.4	99.2±1.7
**3A2T10C**	0.97±0.03	63.2±3.0	1044.4±64.6	100.0±4.2

**Fig 5:**
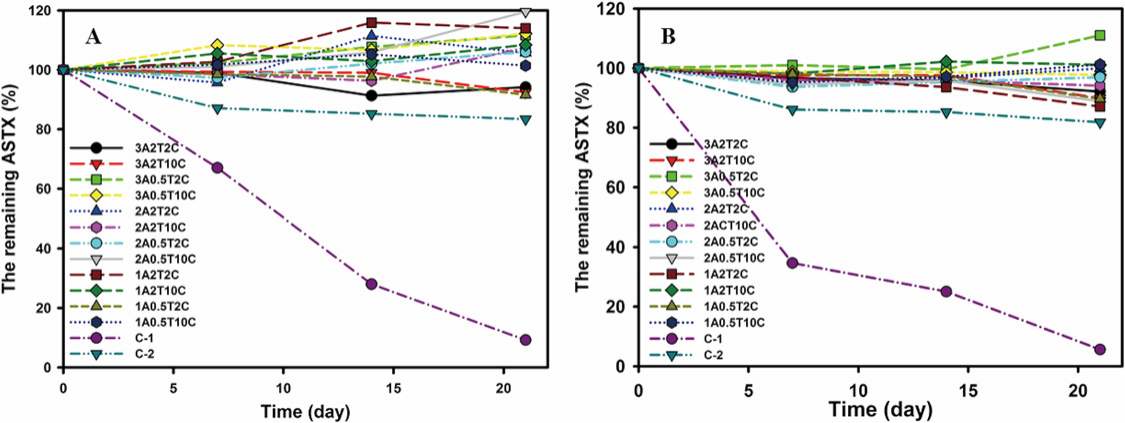
The enhanced stability of astaxanthin encapsulated alginate beads with various formulations under storage conditions at 25°C, RH 60% (**A**) and 40°C, RH 75% (**B**). C-1: the control sample of Carophyll^®^ Pink 10% CWS; C-2: the control sample of pure astaxanthin raw material.

To determine the enhanced stability of encapsulated astaxanthin under different storage conditions (25°C, RH 60%, 40°C, and RH 75%), this study compares the stability of astaxanthin, encapsulated astaxanthin with various formulations, and Carophyll^®^ Pink, a commercial product containing 10% astaxanthin. A pre-study first evaluated the stability of astaxanthin under various environmental factors such as light, nitrogen storage, and temperature. Results show that astaxanthin is most sensitive to high temperature, agreeing with the results of Kittikaiwan et al. [15]. Hence, the storage conditions were set at 40°C and 25°C to test stability. The astaxanthin contents of various formulations of alginate beads and controls were measured periodically during the 21 d of the study. The results in Fig 5 indicate that, regardless of the storage conditions, the content of astaxanthin encapsulated in alginate beads was significantly higher than the two controls (without encapsulation). This can be explained by the reason that the alginate matrix protects the astaxanthin from thermal degradation and blocks the attack of oxygen. Note that the extraction method caused some fluctuations in astaxanthin content during storage, indicating that the technique of extracting astaxanthin from alginate beads can be improved. After 21 d of storage at 25°C, regardless of the formulation used, the content of astaxanthin encapsulated in alginate beads remained more than 90% of the original amount. These results demonstrate that the proposed technique can be used to enhance the stability of other sensitive compounds.

## Conclusions

It is concluded that astaxanthin loaded alginate beads were successfully produced by optimally adjusting formulation factors of alginate, calcium chloride, and Tween 20 concentrations and processing conditions of stirring rate and time, and needle size. The utilization of alginate to encapsulate astaxanthin with the aid of calcium ion was able to significantly improve the thermal stability of astaxanthin. Calcium ion plays dual functional roles as a crosslinker to crosslink alginate chain leading to the formation of alginate matrix layer for preventing oxygen exposure and as a complexer to form the complex with astaxanthin enhancing its stability. Ultimately, the bioactivity of astaxanthin loaded alginate beads needs to be examined to confirm its clinical applicability.

## References

[1] Shahidi F, Synowiecki J (1991) Isolation and characterization of nutrients and value-added products from snow crab (Chinoecetes opilio) and shrimp (Pandalus borealis) processing discards. J Agr Food Chem. 39:1527–1532.

[2] Bhosale P, Bernstein PS (2005) Microbial xanthophylls. Appl. Microbiol. Biot. 68:445–455. 10.1007/s00253-005-0032-8 16001255

[3] Ausich RL (1997) Commercial opportunities for carotenoid production by biotechnology. Pure Appl. Chem. 69:2169–2173.

[4] Higuera-Ciapara I, Felix-Valenzuela L, Goycoolea FM (2006) Astaxanthin: A Review of its Chemistry and Applications. Crit Rev Food Sci Nutr. 46, 185–196. 16431409 10.1080/10408690590957188

[5] Yuan JP, Chen F (2000) Purification of trans-astaxanthin from a high-yielding astaxanthin ester-producing strain of the microalgae Haematococcus pluvialis. Food Chem 68:443–448.

[6] Santocono M, Zurria M, Berrettini M, Fedeli D, Falcioni G (2006) Influence of astaxanthin, zeaxanthin and lutein onDNA damage and repair in UVA-irradiated cells. J. Photochem. Photobiol. B: Biol. 85:205–215. 16962787 10.1016/j.jphotobiol.2006.07.009

[7] Nishino H, Murakoshi M, Xiao YM, Wada S, Masuda M, Ohsaka Y, et al (2005) Cancer prevention by phytochemicals. Oncology 69:38–40. 16210876 10.1159/000086631

[8] Nishigaki I, Dmitrovskii AA, Miki W, Yagi K (1994) Suppressive effect of astaxanthin on lipid peroxidation induced in rats. J. Clin. Biochem. Nutr. 16:161–166.

[9] Yuan C, Jin Z, Xu X, Zhuang H (2008) Preparation and stability of the inclusion complex of astaxanthin with hydroxypropyl-β-cyclodextrin. Food Chem. 109: 264–268 doi: 10.1016/j.foodchem.2007.07.051 26003346 10.1016/j.foodchem.2007.07.051

[10] Kim S, Cho E, Yoo J, Cho E, Choi SJ (2010) *β*-CD-mediated Encapsulation Enhanced Stability and Solubility of Astaxanthin. J. Korean Soc. Appl. Biol. Chem. 53:559–565.

[11] Shahidi F, Han XQ (1993) Encapsulation of food ingredients. Crit Rev Food Sci Nutr 33:501–547. 8216812 10.1080/10408399309527645

[12] Gouin S (2004) Micro-encapsulation: Industrial appraisal of existing technologies and trends. Trends Food Sci Tech. 15:330–347.

[13] Desai KGH, Park HJ (2005) Recent developments in microencapsulation of food ingredients. Dry Technol. 23:1361–1394.

[14] Higuera-Ciapara I, Felix-Valenzuela L, Goycoolea FM (2004) Arguelles-Monal W. Microencapsulation of astaxanthin in a chitosan matrix. Carbohyd. Polym. 56:41–45.

[15] Kittikaiwan P, Powthongsook S, Pavasant P, Artiwan S (2007) Encapsulation of *Haematococcus pluvialis* using chitosan for astaxanthin stability enhancement. Carbohyd Polym. 70:378–385.

[16] Shilpa A, Agrawal SS, Ray AR (2003) Controlled delivery of drug from alginate matrix. J Macromol Sci. C43:187–221.

[17] Martinsen A, Skjak-Braek G, Smidsrod O (1989) Alginate as Immobilization Material: I. Correlation Between Chemical and Physical Properties of Alginate Gel Beads. Biotechnol Bioeng 33:79–83. 18587846 10.1002/bit.260330111

[18] Sugawara S, Imai T, Otagiri M (1994) The controlled release of prednisolone using alginate gel. Pharmaceutical Res 11:272–7. 10.1023/a:1018963626248 8165187

[19] Ribeiro HS, Rico LG, Badolato GG, Schubert H (2005) Production of O/W emulsions containing astaxanthin by repeated premix membrane emulsification. J Food Sci. 70:E117–E123.

[20] Wan LSC, Heng PWS, Chan LW (1992) Drug encapsulation in alginate microspheres by emulsification. J Microencapsulation 9:309–16. 1403481 10.3109/02652049209021245

[21] Shu B, Yu W, Zhao Y, Liu X (2006) Study on microencapsulation of lycopene by spray-drying. J. Food Eng. 76:664–669.

